# Modulation of Kynurenic Acid Production by N-acetylcysteine Prevents Cognitive Impairment in Adulthood Induced by Lead Exposure during Lactation in Mice

**DOI:** 10.3390/antiox12122035

**Published:** 2023-11-23

**Authors:** Paulina Ovalle Rodríguez, Daniela Ramírez Ortega, Tonali Blanco Ayala, Gabriel Roldán Roldán, Gonzalo Pérez de la Cruz, Dinora Fabiola González Esquivel, Saúl Gómez-Manzo, Laura Sánchez Chapul, Aleli Salazar, Benjamín Pineda, Verónica Pérez de la Cruz

**Affiliations:** 1 Neurochemistry and Behavior Laboratory, National Institute of Neurology and Neurosurgery “Manuel Velasco Suárez”, Mexico City 14269, Mexico; paulina.ovalle.rodriguez@gmail.com (P.O.R.); drmz_ortega@hotmail.com (D.R.O.); tblanco@innn.edu.mx (T.B.A.); dinora.gonzalez@innn.edu.mx (D.F.G.E.); 2 Posgrado en Ciencias Bioquímicas, Universidad Nacional Autónoma de México, Ciudad Universitaria, Unidad de Posgrado, Mexico City 04510, Mexico; 3 Laboratorio de Neurobiología de la Conducta, Departamento de Fisiología, Facultad de Medicina, Universidad Nacional Autónoma de México, Mexico City 04510, Mexico; gabaergico@gmail.com; 4 Department of Mathematics, Faculty of Sciences, Universidad Nacional Autónoma de México UNAM, Mexico City 04510, Mexico; gonzalo.perez@ciencias.unam.mx; 5 Laboratorio de Bioquímica Genética, Instituto Nacional de Pediatría, Secretaría de Salud, Mexico City 04530, Mexico; saulmanzo@ciencias.unam.mx; 6 Neuromuscular Diseases Laboratory, Clinical Neurosciences Division, National Institute of Rehabilitation “Luis Guillermo Ibarra Ibarra”, Mexico City 14389, Mexico; lausanchez@inr.gob.mx; 7 Neuroimmunology Department, National Institute of Neurology and Neurosurgery “Manuel Velasco Suárez”, Mexico City 14269, Mexico; aleli.salazar@innn.edu.mx (A.S.); benjamin.pineda@innn.edu.mx (B.P.)

**Keywords:** heavy metals, kynurenic acid, cognition, N-acetylcysteine

## Abstract

Lead (Pb^2+^) exposure during early life induces cognitive impairment, which was recently associated with an increase in brain kynurenic acid (KYNA), an antagonist of NMDA and alpha-7 nicotinic receptors. It has been described that N-acetylcysteine (NAC) favors an antioxidant environment and inhibits kynurenine aminotransferase II activity (KAT II, the main enzyme of KYNA production), leading to brain KYNA levels decrease and cognitive improvement. This study aimed to investigate whether the NAC modulation of the brain KYNA levels in mice ameliorated Pb^2+^-induced cognitive impairment. The dams were divided into four groups: Control, Pb^2+^, NAC, and Pb^2+^+NAC, which were given drinking water or 500 ppm lead acetate in the drinking water ad libitum, from 0 to 23 postnatal days (PNDs). The NAC and Pb^2+^+NAC groups were simultaneously fed NAC (350 mg/day) in their chow from 0 to 23 PNDs. At PND 60, the effect of the treatment with Pb^2+^ and in combination with NAC on learning and memory performance was evaluated. Immediately after behavioral evaluation, brain tissues were collected to assess the redox environment; KYNA and glutamate levels; and KAT II activity. The NAC treatment prevented the long-term memory deficit exhibited in the Pb^2+^ group. As expected, Pb^2+^ group showed redox environment alterations, fluctuations in glutamate levels, and an increase in KYNA levels, which were partially avoided by NAC co-administration. These results confirmed that the excessive KYNA levels induced by Pb^2+^ were involved in the onset of cognitive impairment and could be successfully prevented by NAC treatment. NAC could be a tool for testing in scenarios in which KYNA levels are associated with the induction of cognitive impairment.

## 1. Introduction

Lead (Pb^2+^) is a widely utilized metal in the industry due to its malleability, conductivity, and ductility and usefulness as raw material in countless processes [1,2,3]. Due to the non-biodegradable nature of Pb^2+^, it can accumulate in soil, rivers, lakes, and air, which are the main sources of Pb^2+^ exposure for humans. The World Health Organization established that there is no safe concentration of Pb^2+^ in the blood, as even a low amount of Pb^2+^ has been related to cognitive impairment, particularly in children [4,5,6]. This heavy metal can enter the organism by ingestion, inhalation, and absorption, and once inside, it is distributed to the organs and stored in the teeth and bones [7,8,9,10,11,12]. The central nervous system (CNS) is a target of Pb^2+^ toxicity, leading to long-term consequences [5,13,14]. Several clinical and experimental studies have observed that Pb^2+^ effects depend on the period, duration, and route of metal exposure, being more severe and lasting longer when it occurs during the neurodevelopmental period and/or early life, during which several processes occur, such as neurogenesis, neural migration, differentiation, synaptic pruning, neuronal plasticity, and neuronal connections establishment [15,16,17,18].

Several detrimental effects induced by Pb^2+^ exposure, such as calcium-signaling alteration, energetic homeostasis disruption, oxidative stress, inflammation, and neurotransmitter fluctuations, can trigger long-lasting cognitive impairment [19,20,21,22,23]. We previously proposed that kynurenic acid (KYNA) could play a relevant role in Pb^2+^ neurotoxicity [24]. KYNA is an endogenous metabolite derived from the tryptophan catabolism through the kynurenine pathway (KP), mainly synthesized in astrocytes by the kynurenine aminotransferase II (KAT II) and considered a neuromodulator due its inhibitory effects on NMDA and α-7-nicotinic receptors [25,26,27]. In this context, we previously showed that an increase in brain KYNA levels correlated with long-term memory impairment in adult mice exposed to Pb^2+^ during the lactation period (0–23 PNDs) [24]. Accordingly, several studies have demonstrated that the elevation of brain KYNA levels during gestation or early postnatal life led to memory deficits in adulthood [28,29,30,31]. These studies showed that the stimulation of brain KYNA neosynthesis (administrating its precursor L-kynurenine or inhibiting the long branch of KP) resulted in a reduction in extracellular glutamate levels [27,32,33], while its inhibition induced the opposite effect [26]. Additionally, other important neurotransmitters, such as dopamine, acetylcholine, and gamma-aminobutyric acid, increased when brain KYNA was reduced [25,33,34,35,36,37,38]. Taken together, this experimental evidence demonstrated that the manipulation of endogenous KYNA levels had critical effects on different neurotransmitter systems. However, to our knowledge, the influence of brain KYNA modulation within the context of heavy metal exposure during early life had not been previously described.

In this study, N-acetylcysteine (NAC), a mucolytic and a redox modulator, was used as a tool to regulate KYNA production since it had been previously shown that NAC inhibited KAT II activity in human and rat brain tissues [39,40,41]. It is worth mentioning that NAC had been tested for various neurodegenerative and psychiatric diseases, showing beneficial effects on cognitive performance [42,43,44,45]. Furthermore, several preclinical studies showed NAC effectiveness in ameliorating the brain damage induced by diverse neurotoxic agents; however, its protective effects have always been related to the induction of glutathione (GSH) synthesis promotion as well as to its scavenging and anti-inflammatory properties. In view of previous findings that had shown that the subchronic administration of NAC prevented memory impairment induced by increased brain KYNA levels [46], we aimed to explore whether the inhibitory modulation of brain KYNA levels by NAC administration during early-life Pb^2+^ exposure could prevent cognitive impairments in adulthood.

## 2. Materials and Methods

### 2.1. Materials

L-kynurenine (kyn), kynurenic acid (KYNA), NAC, pyruvate, pyridoxal-5-phosphate (P5P), glutathione reduced form (GSH), oxidized glutathione (GSSG), O-phtaldehyde (OPA), N-Ethylmaleimide (NEM), diethylenetriamine pentaacetate (DTPA), glucose-6-phosphate (G-6P), and glucose-6-phosphate dehydrogenase (G-6PDH) were obtained from Sigma-Aldrich Company (St. Louis, MO, USA). All other chemicals were of the highest commercially available purity. Solutions were prepared using deionized water obtained from a Milli-Q (Millipore, Burlington, MA, USA) purifier system.

### 2.2. Animals 

Female C57 mice were housed 1:1 with a male mouse in individual acrylic cages. Successful mating was confirmed by the presence of mouse sperm on vaginal swabs. Once pregnancy was confirmed, the male was removed from the cage. Dams were housed individually until the day of birth and remained with their pups until weaning at postnatal day (PND) 23. At birth, litters were randomly assigned to one of four groups: (1) Control, (2) Pb^2+^, (3) NAC, and (4) Pb^2+^ + NAC. At weaning, pups were separated by sex and housed by treatment. At PND 60, and once all the treatments were completed, pups were evaluated behaviorally and biochemically.

### 2.3. Lead Exposure and N-acetyl-L-cysteine Treatment

Dams and their offspring were randomly divided into 4 groups: (1) Control group, received normal drinking water; (2) Pb^2+^, received 500 ppm lead acetate in drinking water; (3) NAC, received 350 mg/day of NAC in the food; and (4) Pb^2+^ + NAC, received 500 ppm lead acetate in drinking water and 350 mg/day of NAC in the food. The treatment period was from 0 to 23 PNDs. At weaning, the treatments were withdrawn and replaced with normal drinking water and a normal chow diet. 

### 2.4. Behavioral Test 

#### 2.4.1. Buried Food Location Test (BFLT)

BFLT is an adaptation of the model described by Lehmkuhl et al. for olfactory dysfunction evaluation [47]. This test consists of two sessions: training and memory retrieval testing, both performed in an acrylic box (42 × 30 cm), covered with a 2 cm deep layer of sawdust. Mice were habituated twice to the box for 10 min, 24 h, and 48 h, before training. The box had spatial clues (black geometric figures of 8 × 8 cm, placed at a height of 13 cm in the middle of each side of the box). Mice were also habituated to eat sugary pellets (“fruit loops”) in their home cages for seven days before training in order to eliminate food neophobia. Animals were fasted 24 h before the training session, with ad libitum water access. The training session, or learning phase, consisted of 6 trials (2 min inter-trial interval) and a “0” trial, during which the “fruit loops” were present. In trial “0”, a fruit loop was placed in plain sight on the sawdust in a fixed position, where the mouse could find it and gnaw on it for a few seconds. If mice were unable to find the fruit loop within 180 s in the 0 trial, they were gently guided to it; in each trial, the mice were allowed to gnaw the pellet for at least 5 s. In the next 6 trials, the pellet was buried 1 cm under the sawdust in the same fixed quadrant of the box (the location of the pellet was the same in all trials). At the end of each trial, the animals were returned to their home cage, the testing area was cleaned with a 10% ethanol solution, and the sawdust was removed to eliminate odoriferous marks left by the mouse in the previous trial. After 24 h, long-term memory was evaluated in a retention test where the pellet was no longer present; mice were allowed to freely explore the testing box for 180 s. The time spent reaching the precise target location of the buried food (same location as during the training session) was recorded, and the exploration time spent in the target location quadrant was measured. All sessions were video recorded, allowing offline analysis with tracking software (ImageJ 1.54d version, Bethesda, MD, USA). These results were expressed as the time (in seconds) and distance (in centimeters) to reach the target.

#### 2.4.2. Novel Object Recognition Test (NOR) 

The NOR test is based on a behavioral phenomenon called novelty preference in rodents [48]. NOR test was performed in an acrylic box (42 × 42 × 36 cm) covered with white paper and a 2 cm deep layer of sawdust where mice were habituated for 10 min, 24 h, and 48 h, prior to test. The test consisted of two phases: training and probing. In the training phase, two identical objects (A and A′) were placed at the center of the box, equally spaced. The mice were released into the box, starting from the center of the wall with their backs to the objects, and allowed to explore them for 5 min. The first part of the probe phase (short-term memory evaluation) was carried out two hours after the training session, one of the familiar objects was changed for a novel object “B”, and mice were reintroduced into the box and allowed to explore for another 5 min. The second probe phase to evaluate long-term memory was performed 24 h later in the same way, but object “B” was changed to a novel object “C”. The sessions were recorded, and the exploration time (sniffing and manipulating the objects) was registered. The results were expressed as a recognition index (time exploring a novel object/time exploring both objects × 100). 

### 2.5. Tissue Collection and Treatment 

Immediately after behavioral testing, mice were euthanized by decapitation. Brains were rapidly removed and placed on ice. Brain tissue (20 mg) was treated immediately for GSH-level quantification, and the remaining tissue was rapidly frozen for posterior analysis. To quantify kynurenic acid levels, tissues were homogenized in water (1:10, *w*/*v*), deproteinized with 30 µL of perchloric acid (PCA), and finally, centrifuged at 14,600× *g* for 10 min. 

### 2.6. GSH and GSSG Levels 

Brain tissue was homogenized (1:10, p/v) in Buffer A (154 mM KCl, 5 mM DTPA, and 0.1 M potassium phosphate buffer (PPB) pH 6.8), and then, Buffer B (20 mM ascorbic acid, 10 mM DTPA, 40 mM HCl and 10% trichloroacetic acid) was added (1:1 buffer A). Homogenates were centrifuged at 14,000× *g* for 10 min, and supernatants were filtered (0.22 µm). For GSH determination, OPA was added to the supernatant where it formed an isoindole with GSH that could be fluorometrically detected. To quantify GSSG levels, extra steps were performed: (1) GSH neutralization by mixing supernatants with NEM (7.5 mM), and (2) GSSG levels were reduced to GSH with 100 mM of sodium hydrosulfite; finally, GSSG was detected as GSH for its quantification as an isoindole when adding OPA. The final product was quantified fluorometrically at 370 nm excitation and 420 nm emission in a Synergy HTX microplate reader spectrophotometer (BioTek Instruments, Winooski, VT, USA). GSH and GSSH levels were expressed as nmoles/g of tissue. 

### 2.7. Lipoperoxidation

Brain tissue was used to evaluate lipid peroxidation through thiobarbituric-acid-reactive species (TBA-RS). Briefly, tissues were homogenized in Buffer Krebs (NaCl (19 mM), KCl (5 mM), CaCl_2_ (2 mM), MgSO_4_ (1.2 mM), glucose (5 mM), NaH_2_PO_4_ (13 mM), and Na_2_HPO_4_ (3 mM); pH 7.4). A total of 250 µL of TBA solution (100 mL: 2.54 mL HCl, 0.375 g of TBA, and 15 g of trichloroacetic acid (TCA)) were mixed with 125 µL of homogenate, in a final volume of 500 µL. Then, these homogenates were boiled for 15 min. Subsequently, they were placed on ice for 5 min and centrifugated 12,000× *g* for 5 min at 4 °C. Finally, MDA-TBA chromogen was determined at 532 nm using a microplate reader (Synergy™ HTX, Biotek Instruments, Winooski, VT, USA).

### 2.8. Kynurenic Acid Determination 

KYNA levels were quantified fluorometrically (344 nm excitation and 398 nm emission) in a Series 200a detector (Perkin Elmer, Waltham, MA, USA). A total of 50 µL of supernatants were isocratically eluted at a flow rate of 1 mL/min onto a 3 µm C18 reverse-phase column (ZORBAX Eclipse XDB 5 µm, 4.6 mm × 150 mm; Agilent, Santa Clara, CA, USA), using a mobile phase consisting of 50 mM sodium acetate, 250 mM zinc acetate, and 3% acetonitrile; the pH 6.2. KYNA had a retention time of ~7 min.

### 2.9. Kynurenine Aminotransferase Activity 

KAT II activity was evaluated in brain tissue homogenized 1:10 in homogenization buffer (Tris-base acetate buffer (0.5 M, pH 8), pyridoxal phosphate (P5P, 50 mM), and 2-mercaptoethanol (775 µL)). A total of 100 microliters of homogenates were mixed with 100 µL of the reaction cocktail, consisting of L-Kyn (100 µM), pyruvate (1 mM), and P5P (80 µM) in Tris-acetate buffer (150 mM, pH 7.4), and then incubated for 2 h at 37 °C in a shaking bath. A total of 20 microliters of trichloroacetic acid (50%) plus 1 mL of HCl (0.1 M) were added to samples to stop the reaction. Then, samples were centrifuged at 14,000× *g* for 10 min. KYNA, the enzymatic product of KAT II, was detected by fluorescence, as mentioned previously. 

### 2.10. Glutamate Quantification

Briefly, brain tissue was homogenized in 40 volumes of 85% methanol with a Teflon homogenizer. The homogenates were centrifuged at 4000× *g* for 10 min at 4 °C. The supernatant was used to perform derivatization with OPA (50%/50%). The samples were eluted on a C18, 3 µm column (ZORBAX Eclipse XDB 5 µm, 4.6 × 150 mm; Agilent, Santa Clara, CA, USA) with a mobile phase consisting of 0.29% glacial acetic and 1.5% de tetrahydroflurane at pH 5.9. Glutamate levels were quantified using a reverse-phase HPLC method with a fluorescence detector (Perkin Elmer series 200a, Waltham, MA, USA) at an excitation wavelength of 390 nm and emission wavelength of 460 nm. 

### 2.11. Protein Quantification 

The Lowry method was used to quantify protein levels. Briefly, 10 µL homogenate (1:20 *v*/*v* with water) was used, C solution (1 mL, A + B solutions) was added (A (sodium tartrate (0.2%), Na_2_CO_3_ (2%) and NaOH (0.4%)) and B (Cu(SO_4_)_3_ (0.5%)), and then samples were incubated at room temperature for 10 min. Then, 100 µL of Folin’s solution (50% *v*/*v* with water) was added. Samples were mixed and incubated at room temperature for 30 min. Synergy^TM^ HTX microplate reader (Biotek Instruments, Winooski, VT, USA) was used to determine the absorbance at 550 nm. 

### 2.12. Statistical Analysis

All data were expressed as the mean ± SEM. Comparisons between groups were performed using the Kruskal–Wallis test with Dunn’s test for multiple pairwise comparisons. Pairwise comparisons of novel object-recognition-test data were analyzed using the Wilcoxon signed-rank test. Spearman’s correlation was used to assess the association between variables. Statistical significance was set at *p* < 0.05. All statistics were calculated with Graph Prism 9.1.0. (GraphPad, San Diego, CA, USA). 

## 3. Results

We had previously observed that mice exposure to Pb^2+^ during the lactation period induced cognitive impairment in adulthood, along with a rise in brain KYNA levels [24]. Taking into consideration that NAC is a modulator of KYNA production due to its KAT II inhibitory activity, we decided to test it as a tool to prevent the cognitive impairment induced by this heavy metal. Using the same experimental Pb^2+^ exposure protocol during the lactation period, the strategy consisted of co-administering NAC with Pb^2+^ and then evaluating the cognitive performance, glutamate, and redox environment, as well as the KYNA levels and KAT II activity.

### 3.1. NAC Attenuates Memory Dysfunction Induced by Pb^2+^ Exposure during Early Life

**Figure 1:**
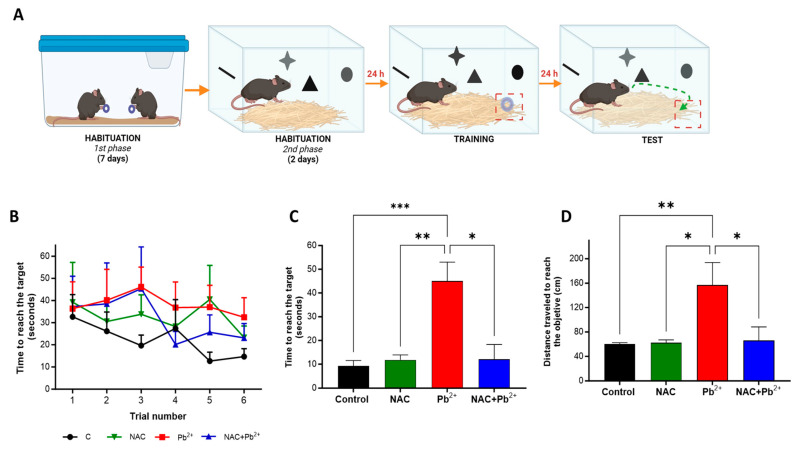
Effect of NAC administration on memory impairment induced by Pb^2+^ exposure. Learning (**B**) and memory (time to reach the target) (**C**) and distance to reach the target (**D**) were evaluated through the buried food location test (**A**) in all experimental groups at 60 PNDs. Data are the mean ± SEM (n = 8–10); * *p* < 0.01, ** *p* < 0.001, and *** *p* < 0.0001, based on the Kruskal–Wallis test with Dunn’s test for pairwise comparisons.

The first step was to use the BFLT to evaluate the cognitive performance of the adult mice (60 PNDs) that were exposed to Pb^2+^ alone or in combination with NAC during the lactation period. The acquisition session (Figure 1A,B) consisted of training mice to learn where the buried food was located. We found no significant differences between the experimental groups during the training session. However, in the Pb^2+^ group, it was observed that the animals showed a less pronounced tendency to learn the target location, since their final time during training was not reduced as markedly as in the other groups, which possibly suggested an impairment in the learning process. Interestingly, the NAC+Pb^2+^ group behaved more similarly to the controls than to the Pb^2+^ group. Furthermore, when long-term memory was evaluated (24 h after training), mice of the Pb^2+^ group took almost the same time (45 ± 8 s) and travel distance to reach the target (156 ± 37 cm) as before training (first trials of the acquisition phase), while the control group had similar times to those observed during the last trials of the acquisition phase (9.3 ± 2.4 s and 59.9 ± 2.5 cm, respectively), confirming that the early postnatal exposure to this heavy metal induced cognitive impairment. The NAC group showed no changes, as compared to the control group, when assessing long-term memory. For those administered with Pb^2+^ and NAC simultaneously, they showed improved long-term memory performance by reducing the time and distance required to reach the target (12 ± 2.2 s and 65.7 ± 8.5 cm, respectively), as compared to the Pb^2+^ group (Figure 1C and Figure 1D, respectively).

**Figure 2:**
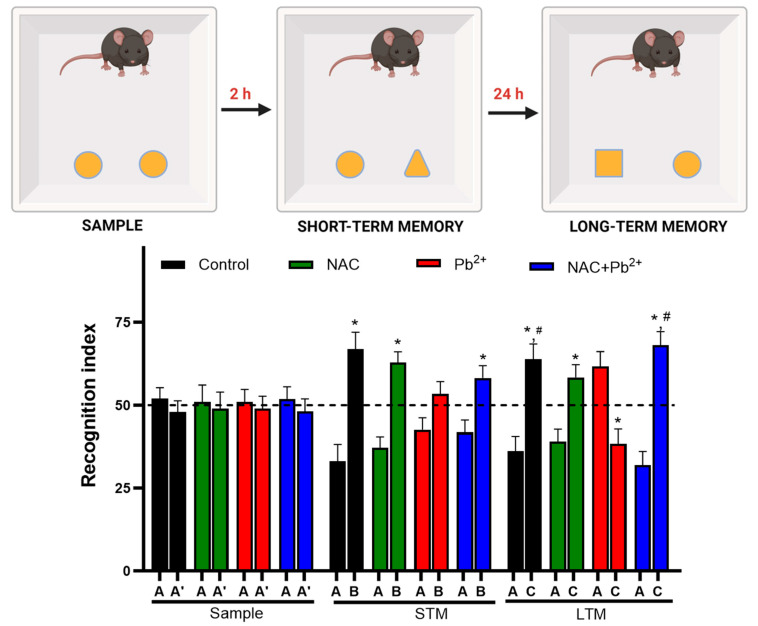
Effect of simultaneous administration of NAC and Pb^2+^ during lactation on the evaluation of short-term memory (STM) and long-term memory (LTM) through novel object recognition test. The recognition index (time exploring novel object/time exploring both objects × 100) was calculated for 8–10 animals per group. The data represent mean ± SEM; * *p* < 0.05 between the novel and familiar objects for each group and phase based on the Wilcoxon signed-rank test; and *^#^ p* < 0.001 vs. Pb^2+^ based on the Kruskal–Wallis test with Dunn’s test for pairwise comparisons.

The novel object recognition (NOR) test was another strategy used to evidence the NAC effect on the cognitive impairment induced by Pb^2+^ exposure. This test was based on the innate preference of the mice for novelty [49]. We first confirmed that mice from all the groups spent an equal amount of time exploring two identical objects, A and A′ (Figure 2, sample phase). Next, when the short-term memory was evaluated, all groups except for the Pb^2+^ group showed that the mice were able to distinguish the novel object from the familiar one, while the Pb^2+^ group was unable to discriminate between both objects though they had the same exposure in the sample phase (Figure 2, short-term memory (STM)). When the long-term memory (LTM) was evaluated, as expected, the control group explored the novel object for longer periods of time than the familiarized object as evidenced by a significant increase in the discrimination index, while the Pb^2+^ group could not discriminate between objects (Figure 2, LTM). The long-term memory impairment induced by Pb^2+^ exposure was abolished by the simultaneous administration of NAC, indicating that NAC had prevented deficits in long-term memory. 

### 3.2. Fluctuations in Brain KYNA Levels Are Prevented by NAC Administration

**Figure 3:**
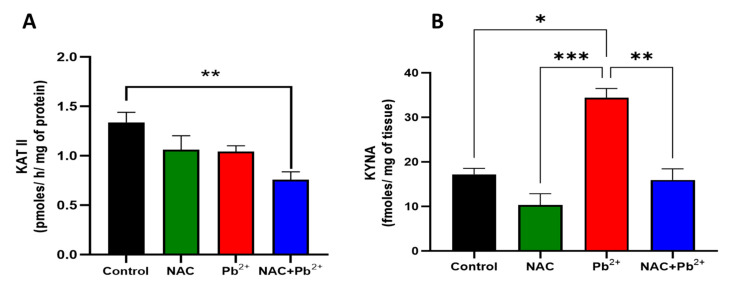
Effect of simultaneous administration of NAC and Pb^2+^ on KAT II activity and brain KYNA levels in mice at 60 PNDs. NAC and Pb^2+^ were administrated during the lactation period; after that, the animals were administrated tap water and a standard mice diet until 60 PNDs. The brain cortex was used to evaluate KAT II activity (**A**) and KYNA levels (**B**). Data represents mean ± SEM of 7–10 animals per group. * *p* < 0.01, ** *p* < 0.001, and *** *p* < 0.0001 based on the Kruskal-Wallis test with Dunn’s test for pairwise comparisons.

As previously described, the cognitive impairment induced by the Pb^2+^ administration correlated with an elevation of the brain KYNA levels; here, we aimed to examine whether NAC could prevent Pb^2+^-induced cognitive impairment by reducing the brain KYNA levels inhibiting KAT II activity. Therefore, we first determined the effect of NAC on the KYNA levels and the KAT II activity in those adult mice (60 PNDs) that were exposed to Pb^2+^ during lactation. As shown in Figure 3A, no significant changes in the KAT II activity were observed in the NAC and Pb^2+^ groups, as compared to the controls. Conversely, in the NAC+Pb^2+^ group, a significant reduction (around 40%) in the KAT II activity was found in the brain tissue. As expected, in the Pb^2+^ group, the brain KYNA levels increased (about 2-fold), as compared to the control group, whereas a non-significant reduction was found in the brain KYNA levels in the NAC group. Furthermore, the increase in the brain KYNA levels induced by Pb^2+^ exposure was prevented by NAC treatment, and this effect could have been partially due to the KAT II inhibition shown in the same group.

**Figure 4:**
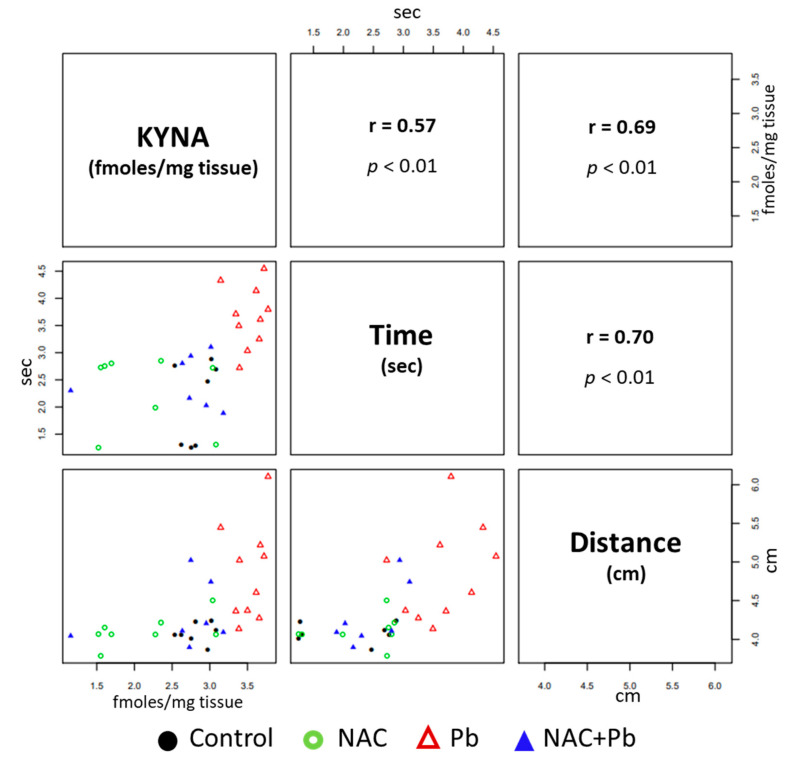
Correlation between long-term memory and brain KYNA levels. The lower triangular matrix contains the scatterplot for each pair of variables for all groups (Control: black circle; NAC: green circle; Pb^2+^: red triangle; and NAC+Pb^2+^: blue triangle). The upper triangular matrix contains the Spearman’s rank correlation coefficient (r) and its associated *p*-value (*p*) (n = 7–10 per group).

Based on our data that NAC could prevent the cognitive impairment induced by Pb^2+^, we speculated if the pro-cognitive effect exerted by NAC on Pb^2+^-toxicity was directly correlated to the brain KYNA-level modulation. To answer this question, we analyzed the learning and memory performance parameters (time and distance to reach the target) obtained from the BFLT results of all the groups with their respective brain KYNA levels (Figure 4). A positive association between the brain KYNA levels and the long-term memory performance was found, thus suggesting that NAC improved cognitive performance, at least in part, by modulating the brain KYNA levels.

### 3.3. Effect of NAC Co-Administration on Brain Glutamate Level Fluctuations Induced by Pb^2+^ Exposure 

**Figure 5:**
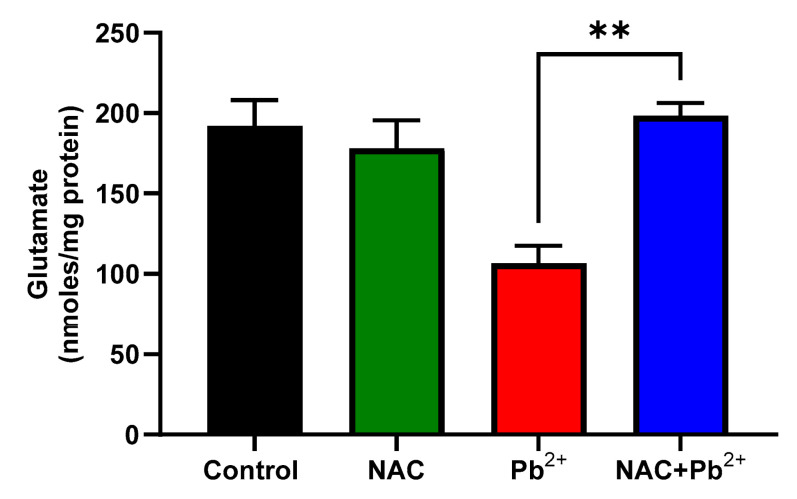
Effect of NAC on glutamate levels when exposed to Pb^2+^ during lactation. Brain levels of glutamate were evaluated in all the experimental groups at 60 PNDs. Data represent mean ± SEM of 4–5 animals per group. ** *p* < 0.001 based on the Kruskal-Wallis test with Dunn’s test for pairwise comparisons.

We had confirmed the involvement of KYNA as a part of the mechanisms by which Pb^2+^ exposure induces cognitive impairment. We also knew that this metabolite was a neuromodulator of glutamatergic transmission since previous in vivo studies had indicated that in the hippocampus and prefrontal cortex, where KYNA had decreased glutamate levels, presumably via the inhibition of α7 nicotinic receptors (nAChRs) [32,33]. As shown in Figure 5, the Pb^2+^ group showed a trend of decreasing glutamate levels while NAC itself did not have any effect on the glutamate levels. Interestingly, the simultaneous administration of NAC with Pb^2+^ exposure had increased the glutamate levels, as compared to the Pb^2+^, suggesting that NAC had prevented a reduction in the marginal glutamate levels induced by Pb^2+^.

### 3.4. Redox Environment Alteration Induced by Pb^2+^ Exposure Is Prevented by NAC Administration

**Figure 6:**
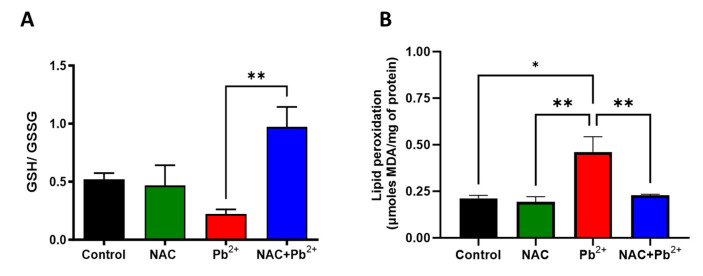
Effect of NAC in the pro-oxidant environment induced by Pb^2+^. Brain levels of GSH/GSSG ratio (**A**) and lipid peroxidation (**B**) were evaluated in all the experimental groups at 60 PNDs. Data represent mean ± SEM of 4–5 animals per group. * *p* < 0.01 and ** *p* < 0.001 based on Kruskal-Wallis test with Dunn’s test for pairwise comparison.

In addition, since the KYNA formation could be carried out via non-canonical pathways that involved a cellular redox state, we expected that the pro-oxidant effects of Pb^2+^ would increase the amount of ROS during Pb^2+^ administration and, confirming our previous data, induce non-enzymatic KYNA production [50,51]. Comparatively, here, our hypothesis was that NAC treatment, given its antioxidant profile during Pb^2+^ administration, would reduce the concentration of these ROS and, thus, the non-enzymatic production of KYNA. To address this objective and considering the short half-life of these ROS, we assessed a GSH/GSSH ratio and lipid peroxidation levels across all experimental groups (Figure 6A and Figure 6B, respectively). The GSH/GSSG ratio was marginally reduced in the Pb^2+^ group (around 38%) while the simultaneous administration of Pb^2+^ with NAC increased the GSH/GSSG ratio, as compared to the Pb^2+^ group. Upon evaluating the lipid peroxidation, a prominent oxidative stress marker, the Pb^2+^ group exhibited an approximately twofold increase, as compared to the control group. However, this oxidative upsurge was effectively neutralized when NAC was administered in conjunction with Pb^2+^, underscoring NAC’s potential in mitigating the Pb^2+^-induced oxidative stress and its consequent impact on the cellular redox environment and the non-enzymatic KYNA production.

## 4. Discussion

The goal of the present study was to investigate whether the NAC modulation of the brain KYNA levels could mitigate the cognitive impairment induced by Pb^2+^ exposure during early postnatal life. This approach on the modulation of brain KYNA levels was based on our previous findings that had shown a correlation between a pronounced increase in the brain KYNA levels and the Pb^2+^-induced long-term memory impairment during the lactation period, in a mouse model [24]. 

The important role of KYNA on neurotransmission has been extensively demonstrated in different rodent models; hence, we now know that KYNA influences GABAergic, cholinergic, glutamatergic, and dopaminergic neurotransmission [25,36,37,38,52]. However, among this range of neuromodulatory functions, the effect of KYNA on glutamatergic neurotransmission is the most thoroughly understood. Experimental evidence has shown that fluctuations in the brain KYNA levels substantially reduced the extracellular glutamate levels in the different brain areas, including those related to cognitive processes, such as the hippocampus and the prefrontal cortex [27,32,33]. In fact, KYNA has been used as a tool in several experimental models to reduce or block glutamate-mediated neurotransmission. Specifically, experimental manipulations to increase the brain levels of KYNA have shown that nanomolar or low micromolar elevations of this metabolite induced a wide range of cognitive impairments, including disrupting hippocampus-mediated contextual learning and memory; working memory; and contextual fear memory [32,53]. In addition to pharmacological manipulations, some exogenous stimuli have been known to induce an increase in the brain KYNA levels. Similarly, we previously demonstrated that Pb^2+^ exposure during lactation had increased the brain levels of KYNA when evaluated at 23 PNDs and 60 PNDs [24]. Our data suggested that the elevation in the KYNA levels could be a mechanism by which Pb^2+^ exposure during lactation induced cognitive impairment in adult mice (60 PNDs). Thus, if the elevated brain KYNA levels were a key mechanism in the induction of cognitive deficits, reducing the KYNA levels in this same model could prevent the observed alterations in learning and memory during adulthood.

To reduce the KYNA levels, we decided to use NAC as a pharmacological tool, as this compound had been previously shown to inhibit the main enzyme for KYNA synthesis, KAT II [39]. Therefore, to address the effect of reducing the KYNA levels by NAC on Pb^2+^-induced cognitive deficits, we first confirmed that Pb^2+^ induced long-term memory alterations in adult mice when exposed during lactation. As expected, when NAC was co-administered with Pb^2+^, it successfully mitigated the Pb^2+^-induced cognitive impairment in both cognitive tests performed. The next question was to investigate whether this cognitive improvement could be related to a reduction in the brain KYNA levels modulated by NAC. 

Under our experimental conditions and as we had previously shown, Pb^2+^ exposure during lactation induced a substantial increase in KYNA levels when the brain tissue was examined after the cognitive testing at 60 PNDs. This increase was successfully prevented by the NAC administration, as shown in the brain tissue of those animals co-administered with NAC during Pb^2+^ exposure. In this group, KAT II activity was significantly reduced in the brain tissue; this could partly explain the reduction in the KYNA levels. Moreover, when we analyzed Pb^2+^ and NAC groups separately, no significant effect on the KATII activity was shown [24]. In the case of the NAC+Pb^2+^ group, the potential explanation for our findings was that NAC had previously been demonstrated to reduce glial cell activation, mainly reactive astrocytes, which were the major cells expressing KAT II [54]. Accordingly, in cultured astrocytes, it has been shown that GFAP, a protein marker for reactive astrocytes, was not as overexpress after Pb^2+^ treatment as it was in response to Pb^2+^-induced neuronal damage, indicating that astrogliosis was more likely a secondary reaction to this event [55]. Therefore, if neuronal damage was prevented, astrogliosis would also be reduced. Additionally, NAC has been observed to promote neuronal differentiation, thereby potentially improving the neuron/glia ratio, which could have subsequent effects on the KAT II activity due to its cellular distribution [56]. In the NAC-only group, it was also important to consider that the observed effect on the KAT II activity was measured 30 days after the cessation of the NAC treatment; we could not rule out a direct inhibitory effect on KAT II in this experimental group during the NAC administration (lactation). Future experiments addressing the activity of this enzyme at different times of the treatment should be performed to determine the mechanism of the KYNA reduction by NAC more precisely under these basal conditions. 

Therefore, these results not only confirmed that the NAC administration during the lactation period could efficiently prevent an increase in the brain KYNA levels induced by Pb^2+^ exposure, but that this effect was also sustained into adulthood, where it translated into the ameliorating cognitive impairments observed in the Pb^2+^ group. This was consistent with the experimental evidence showing that KAT II inhibition, pharmacologically or by genetic manipulation (i.e., KATII knockout mice), had pro-cognitive effects [32,57]. Moreover, consistent with our findings, it was reported that NAC supplementation had prevented cognitive impairment associated with transient increases in the brain KYNA levels [46]. 

The detrimental cognitive effects of increased brain KYNA during gestational or early postnatal stages have been believed to stem from its ability to modulate glutamatergic neurotransmission [29]. Glutamate, the predominant excitatory neurotransmitter in the CNS, mediated 70–90% of synaptic transmission and played a vital role in learning and memory, as well as synaptic plasticity [58,59]. As mentioned previously, there has been plenty of evidence suggesting that elevated brain KYNA reduced glutamate levels (around 30–50%) [27,32,33], as observed in this study where postnatal Pb^2+^ exposure resulted in elevated brain KYNA levels while the glutamate in the brain tissue decreased. The NAC administration had normalized both the brain KYNA and glutamate fluctuations induced by Pb^2+^. Our results also confirmed that the pro-cognitive effects of the KYNA reduction induced by NAC preserved the glutamate levels in the brain tissue, thus allowing the prevention of the alterations in neurotransmission and, consequently, in the cognitive performance impairments related to the excessive Pb^2+^-induced KYNA production. On the other hand, it is noteworthy to mention that most of the pro-cognitive experimental manipulations of KYNA had been described under physiological conditions. Here, we described that the reduction in the KYNA levels, even within the context of Pb^2+^-induced neurotoxicity, could be beneficial for cognitive processes. Furthermore, if we considered that the detrimental effects of Pb^2+^ exposure on the CNS have been well documented, particularly when exposure had occurred in early life, which is a critical period for establishing proper communication between brain cells [60,61,62], the pro-cognitive effect induced by the KYNA manipulation in the lactation period during Pb^2+^ exposure was more relevant. This kind of modulation could also potentially be translatable to humans since the prenatal concentrations of KYNA in the human brain are much higher than during adulthood, suggesting a key role as a direct modulator of glutamatergic neurotransmission in the developing brain [63].

Separately, we should mention that NAC is also a modulator of the redox environment [64,65]. This antioxidant profile was relevant within the context of non-canonical production of KYNA, where its synthesis was promoted by the direct interaction of its precursor, L-kynurenine, with ROS and free radicals [50]. Given that Pb^2+^ exposure has been known to promote oxidative stress by increasing ROS and disrupting the antioxidant balance, it is conceivable that KYNA could be produced via this non-canonical pathway. To verify whether these pro-oxidants conditions were promoting this pathway, we evaluated markers of oxidative stress, including lipid peroxidation and a GSH/GSSG ratio. As anticipated, NAC prevented the Pb^2+^-induced lipid peroxidation and significantly increased the GSH/GSSG ratio, as compared to the Pb^2+^ group. These findings suggested that NAC helped to mitigate the ROS levels, consequently reducing the likelihood of KYNA being produced through the non-canonical pathway. 

In this context, it had been observed that the pre-treatment with NAC before exposure to a pro-oxidant agent enabled brain tissue to better withstand oxidative damage, as compared to when no supplementation had been provided [46]. This indicated that subchronic NAC supplementation may prime or adapt the brain environment to better cope with subsequent adverse effects. Consistent with this, the simultaneous administration of NAC and Pb^2+^ resulted in reduced brain malondialdehyde levels (around 54% vs. Pb^2+^), the upregulation of SOD gene expression, the downregulation of cell-death-related genes, and the improvement of the GSH/GSSG ratio [66,67]. Furthermore, in vitro studies had demonstrated that NAC had the capacity to bind Pb^2+^ [68,69]. This interaction may have contributed to the elimination of Pb^2+^ through feces and urine [70], providing another mechanism through which NAC exerted its protective effects against Pb^2+^-induced oxidative stress and the subsequent KYNA production.

It is crucial to note that both cognitive performance and biochemical test were evaluated as the long-term effects of early-life KYNA modulation. As we mentioned previously, early-life Pb^2+^ exposure disrupts several essential brain processes [60,61,62]. Previous studied had shown that mice exposed to Pb^2+^ during early life exhibited increased brain KYNA levels and, later, demonstrated cognitive impairments in adulthood [24]. This suggested that even mild increases in the brain KYNA levels, initiated by Pb^2+^ exposure in the earliest postnatal days, may interfere with the establishment of neural networks. In our study, the NAC supplementation prevented the Pb^2+^ -induced increases in the brain KYNA, correlating with improved cognitive performance following Pb^2+^ exposure. These findings indicated that KYNA production was a mechanism through which Pb^2+^ induced neurotoxicity and highlighted KYNA modulation as a significant pathway through which NAC exerted its pro-cognitive effects. While numerous studies have underscored the protective effects of NAC in cognitive dysfunction across various experimental models and human pathologies, often attributing these benefits to its antioxidant or anti-inflammatory properties [71,72,73,74], our study confirmed KYNA modulation as a crucial mechanism [46], even in a pro-oxidant context, underlying NAC’s pro-cognitive effects.

As detailed in this paper, numerous studies have sought to unravel the effects of NAC on Pb^2+^ neurotoxicity [67,75,76,77]. These studies collectively highlighted NAC’s ability to improve cellular redox status, mitigate inflammatory responses, improve mitochondrial bioenergetics, support neurotransmission, and protect against Pb^2+^-induced neuronal cell death. Consequently, a pertinent question arose: could the KYNA modulation observed in this study be a secondary effect of NAC’s influence on these various factors? In our research, we have established a direct correlation between KYNA levels and cognitive outcomes, underscoring KYNA’s significant role in the neurotoxicity triggered by Pb^2+^ exposure. Given that NAC has demonstrated efficacy in counteracting the factors that influence KYNA formation, it is plausible that NAC could hinder certain pathways leading to the synthesis of this tryptophan metabolite. However, it is important to acknowledge a limitation of our study: NAC is not a specific inhibitor of KAT II. This non-specificity makes it challenging to definitively ascertain how NAC precisely modulates only brain KYNA levels. Despite this limitation, our findings clearly indicated that NAC reduced KYNA levels, and this reduction was associated with cognitive improvements in the presence of Pb^2+^. Another notable limitation was that all the parameters evaluated in this study were determined long-term, at 60 PNDs, following the cessation of both the Pb^2+^ exposure and the NAC treatment, at 23 PND. Future studies should aim to investigate KYNA modulation’s impact, both during and immediately after Pb^2+^ exposure.

## 5. Conclusions

The comprehensive results of our study underscored the pivotal role of brain KYNA in mediating cognitive impairments induced by Pb^2+^ exposure during early life. We have established a direct correlation between elevated KYNA levels and compromised cognitive function, highlighting the neurotoxic potential of this tryptophan metabolite when dysregulated by heavy metal exposure. Also, our findings pave the way for further exploration of NAC as a multifaceted therapeutic agent, particularly in scenarios where dysregulated KYNA levels are implicated in cognitive impairments. The potential for NAC to modulate KYNA production, alongside its well-documented antioxidant and anti-inflammatory properties, positions it as a valuable candidate for intervention in a spectrum of cognitive disorders.

## Data Availability

Data used to support the findings of this study are available from the corresponding author upon request.
